# Sleep Abnormalities in the Synaptopathies—*SYNGAP1*-Related Intellectual Disability and Phelan–McDermid Syndrome

**DOI:** 10.3390/brainsci11091229

**Published:** 2021-09-17

**Authors:** Constance Smith-Hicks, Damien Wright, Aisling Kenny, Robert C. Stowe, Maria McCormack, Andrew C. Stanfield, J. Lloyd Holder

**Affiliations:** 1 Division of Neurogenetics Kennedy Krieger Institute, 1741 Ashland Avenue Rm 526, Baltimore, MD 21205, USA; 2 Department of Neurology, Johns Hopkins University School of Medicine, Baltimore, MD 21287, USA; 3 Patrick Wild Centre, University of Edinburgh, Edinburgh EH8 9YL, UK; damien.wright@ed.ac.uk (D.W.); akenny3@exseed.ed.ac.uk (A.K.); Andrew.Stanfield@ed.ac.uk (A.C.S.); 4 Department of Neurology, Division of Epilepsy and Clinical Neurophysiology, Boston Children’s Hospital, Boston, MA 02115, USA; robert.stowe@childrens.harvard.edu; 5 Division of Sleep Medicine, Harvard Medical School, Boston, MA 02115, USA; 6 Jan and Dan Duncan Neurological Research Institute, Texas Children’s Hospital 1250 Moursund, Suite 925, Houston, TX 77030, USA; maria.mccormack@bcm.edu; 7 Departments of Pediatrics and Neurology, Baylor College of Medicine, Houston, TX 77030, USA

**Keywords:** Phelan–Mcdermid syndrome, *SYNGAP1*, Children’s Sleep Habits Questionnaire, polysomnography

## Abstract

Neurodevelopmental disorders are frequently associated with sleep disturbances. One class of neurodevelopmental disorders, the genetic synaptopathies, is caused by mutations in genes encoding proteins found at the synapse. Mutations in these genes cause derangement of synapse development and function. We utilized a validated sleep instrument, Children’s Sleep Habits Questionnaire (CSHQ) to examine the nature of sleep abnormalities occurring in individuals with two synaptopathies—Phelan–McDermid syndrome (PMD) (N = 47, male = 23, female = 24, age 1–46 years) and *SYNGAP1*-related intellectual disability (*SYNGAP1*-ID) (N = 64, male = 31, female = 33, age 1–64 years), when compared with unaffected siblings (N = 61, male = 25, female = 36, age 1–17 years). We found that both PMD and *SYNGAP1*-ID have significant sleep abnormalities with *SYNGAP1*-ID having greater severity of sleep disturbance than PMD. In addition, sleep disturbances were more severe for PMD in individuals 11 years and older compared with those less than 11 years old. Individuals with either disorder were more likely to use sleep aids than unaffected siblings. In conclusion, sleep disturbances are a significant phenotype in the synaptopathies PMD and *SYNGAP1*-ID. Improved sleep is a viable endpoint for future clinical trials for these neurodevelopmental disorders.

## 1. Introduction

Neurodevelopmental disorders (NDDs) are severe clinical consequences of abnormal brain development [1]. The phenotypic manifestations of NDDs include sleep disorders, learning disabilities, delayed ascertainment of developmental milestones, intellectual disabilities, behavioral disturbances and epilepsy among others. NDDs can have multiple etiologies including sequelae of infections of the central nervous system either in utero or perinatally, restricted blood flow and oxygenation due to perinatal stroke or perinatal head trauma [2,3]. Increasingly, genetic abnormalities are recognized as a significant cause of neurodevelopmental disorders. These genetic abnormalities include chromosomal derangements such as Trisomy 21, contiguous gene deletion syndromes or single gene disruptions [4,5].

Among all genetic NDDs, synaptopathies are unique in that they are caused by mutations in genes which encode proteins that function at the synapse [6]. The results of these mutations are dysfunctional synapses or alterations in synapse numbers and abnormalities in neuronal network development with consequent neuropsychiatric phenotypes [7]. Two of the most commonly identified synaptopathies, Phelan–McDermid syndrome (PMD) and *SYNGAP1*-related intellectual disability (*SYNGAP1*-ID) have characteristic neurodevelopmental deficits including intellectual disability, global developmental delay, autism and epilepsy [8,9,10,11,12].

Phelan–McDermid syndrome most commonly results from a deletion of chromosome 22q13 encompassing the *SHANK3* gene and variably other genes [13]. In addition, single nucleotide variants in *SHANK3* have also been identified to cause PMD [11]. SHANK3 is a scaffolding protein of the post-synaptic density of excitatory synapses. *SYNGAP1*-related intellectual disability results most commonly from single nucleotide variants that cause loss-of-function mutations [10,12] affecting SynGAP protein which functions as a small GTPase activating protein [14]. SHANK3 and SynGAP are enriched in the post-synaptic density of excitatory neurons where they play important roles in the regulation of homeostatic synaptic plasticity [15]. Intriguingly, sleep is posited to regulate homeostatic plasticity [16] where sleep promotes scaling down of synaptic strengths while wakefulness promotes increase in synaptic strengths. Knockdown of Shank3 in preclinical studies completely abolished synaptic scaling up [15] while knockdown of SynGAP protein led to increases in synaptic strength [17].

Sleep problems have been reported in patients with PMD and *SYNGAP1*-ID [9,10,11,12]; however, most of these have been limited, qualitative descriptions. For *SYNGAP1*-ID, in the largest cohort so far reported [10], 34/55 (61.8%) of individuals with pathogenic *SYNGAP1* mutations had sleep disturbances. Of those with reported sleep disturbances, 19 had difficulty initiating sleep and 29 had difficulty maintaining sleep; however, no structured sleep instrument was used in this study. For Phelan–McDermid syndrome, one study has utilized the Childhood Sleep Habits Questionnaire (CSHQ) to systematically evaluate for sleep disturbances in this population [18]. Overall, 89.9% of individuals with PMD were found to have a sleep disturbance. However, comparison of total score and subscale scores was limited to historical controls.

Structured measures of sleep disturbance have not been systematically applied to *SYNGAP1*-ID nor has there been a comparison of sleep abnormalities between patients with synaptopathies such as *SYNGAP1*-related ID and PMD. Given the inverse relationship between SHANK3 and SynGAP as it relates to homeostatic scaling, we hypothesize that the sleep profile in *SYNGAP1*-ID would be different than that seen in PMD.

We utilized a standardized instrument, the Children’s Sleep Habits Questionnaire (CSHQ), to identify sleep abnormalities manifesting as insomnia, hypersomnia, parasomnias or circadian dysregulation in these two disorders.

## 2. Methods

### 2.1. Recruitment

Participants for this study were recruited from the Bluebird Circle Clinic for Pediatric Neurology at Texas Children’s Hospital, the Kennedy Krieger Institute and the Patrick Wild Centre at the University of Edinburgh with the assistance of family advocacy foundations: Phelan McDermid Syndrome Foundation, Bridge the Gap: *SYNGAP1* Education and Research Foundation and the SynGap Research Fund, Inc. We provided the patient advocacy organizations with a recruitment letter that was sent by email to their registrants. Adult caregivers of individuals from age 12 months and above with Phelan–McDermid syndrome, *SYNGAP1*-related intellectual disability or unaffected siblings were eligible to participate. Exclusionary criteria included brain trauma, brain surgery or hearing impairment for both affected individuals and unaffected siblings as these might impair sleep independently of the genetic disorder. Unaffected siblings did not have a known history of a neurogenetic disorder. Those interested in participating contacted the research staff and informed consent was obtained. The research staff either directly asked the caregivers 33 total questions on the Children’s Sleep Habit Questionnaire (CSHQ) [19] or the caregivers completed an online or paper version of the CSHQ. This instrument assesses parental reports of frequency of various sleep behaviors during a typical week as ‘usually’ (5–7 times/week), ‘sometimes’ (2–4 times/week) or ‘rarely’ (0–1 times/week); higher scores indicate worse sleep problems or behaviors. A total score of greater than 41 is used to indicate the presence of sleep abnormalities. The answers to the CSHQ are then grouped into subscales: bed-time resistance, sleep onset delay, sleep duration, sleep anxiety, night awakenings, parasomnias, sleep disordered breathing and daytime sleepiness. Although CSHQ was originally developed for school age children, it has been previously been used for children with intellectual disabilities of ages beyond 11 years including for Phelan–McDermid syndrome [18].

Answers to the questionnaire were recorded once and was completed in 15–20 min. The caregivers were also asked for typically developed siblings to participate as healthy control individuals. The surveys were performed between May 2019 and June 2021.

Ethical approval was obtained from the Institutional Review Board for Baylor College of Medicine and Affiliated Hospitals (H-44480), the Johns Hopkins University School of Medicine (IRB00188402) and Scotland A Research Ethics Committee (19/SS/0036).

### 2.2. Demographics

Table 1 lists the demographics of the populations recruited for this study. There was no significant difference in age of the participants with *SYNGAP1*-related intellectual disability and unaffected siblings whereas the mean age of participants with Phelan–McDermid syndrome was greater than unaffected siblings. There was no significant difference in sex among the three groups of participants.

### 2.3. Data and Statistical Analysis

Raw data were aggregated in Microsoft Excel. These data were then both summed to determine the total score per individual or parsed into eight sub-scales: bedtime resistance, sleep anxiety, sleep-onset delay, night awakenings, parasomnias, daytime sleepiness, sleep disordered breathing and sleep duration for calculating sub-scale scores per each individual. Data were also parsed and analyzed for those less than 11 years and those 11 years or greater (*SYNGAP1*-ID N = 49 < 11 years, N = 15 ≥ 11 years; PMD N = 24 < 11 years, N = 23 ≥ 11 years; unaffected siblings N = 37 < 11 years, N = 24 ≥ 11 years). This sub-analysis was performed because (1) the CSHQ was initially validated for those less than 11 years of age and (2) to capture any differences in pre-pubertal versus post-pubertal age. Statistical analysis for comparison of PMD, *SYNGAP1*-ID and unaffected sibling data was performed in GraphPad Prism version 8. For comparing all three groups, Kruskal–Wallis non-parametric tests were used. For pairwise comparisons, Dunn’s multiple comparison tests were performed.

## 3. Results

**Figure 1:**
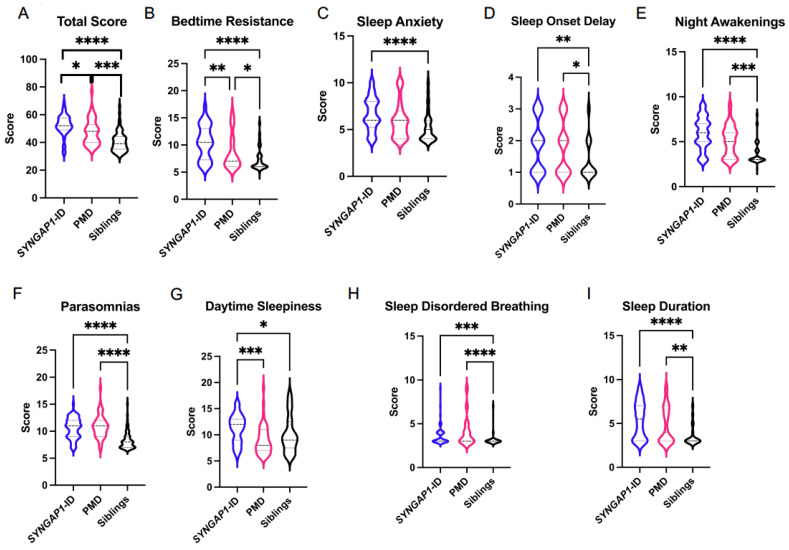
Total (**A**) and sub-scale scores for *SYNGAP1*-related intellectual disability (*SYNGAP1*-ID), Phelan–McDermid syndrome (PMD) and unaffected siblings (Siblings). Sub-scales: (**B**) Bedtime Resistance, (**C**) Sleep Anxiety, (**D**) Sleep Onset Delay, (**E**) Night Awakenings, (**F**) Parasomnias, (**G**) Daytime Sleepiness, (**H**) Sleep Disordered Breathing, (**I**) Sleep Duration. Dunn’s multiple comparisons test * *p* < 0.05, ** *p* < 0.01, *** *p* < 0.001, **** *p* < 0.0001. Heavy dashed line is median, light dashed line is quartile.

The CSHQ average total score (Figure 1 and Table 2) for *SYNGAP1*-ID (M = 52.2 ± 7.7) was significantly increased (*p* < 0.0001) compared with unaffected siblings (M = 40.5 ± 7.4). In contrast, there was a smaller average increased total score (M = 47.6 ± 9.2) for PMD. We then evaluated sub-scale scores between *SYNGAP1*-ID or PMD and unaffected siblings. Participants with *SYNGAP1*-ID had significantly elevated scores on all sub-scales compared with unaffected siblings (Figure 1 and Table 2). For PMD, scores were significantly elevated compared with siblings on all sub-scales except sleep anxiety and daytime sleepiness (Figure 1 and Table 2). We also evaluated whether the participant’s sex impacted sleep scores. Total scores for each disorder as well unaffected siblings did not significantly differ based upon sex (Supplemental Table S1). Thus, no further analysis based upon sex was performed.

**Figure 2:**
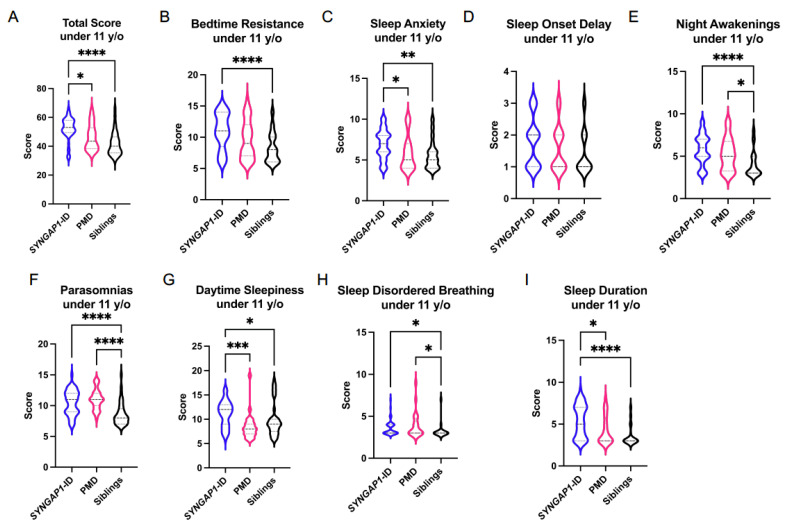
Total (**A**) and sub-scale scores for *SYNGAP1*-related intellectual disability (*SYNGAP1*-ID), Phelan–McDermid syndrome (PMD) and unaffected siblings (Siblings) under 11 years of age. Sub-scales: (**B**) Bedtime Resistance, (**C**) Sleep Anxiety, (**D**) Sleep Onset Delay, (**E**) Night Awakenings, (**F**) Parasomnias, (**G**) Daytime Sleepiness, (**H**) Sleep Disordered Breathing, (**I**) Sleep Duration. Dunn’s multiple comparisons test * *p* < 0.05, ** *p* < 0.01, *** *p* < 0.001, **** *p* < 0.0001. Heavy dashed line is median, light dashed line is quartile.

We next parsed our data into those individuals under 11 years of age and those 11 years old or older to determine if the sleep disorders identified in these synaptopathies are age dependent (Figure 2 and Table 3). For those individuals under 11 years old, we again found *SYNGAP1*-ID individuals have a total score (M = 52.2 ± 7.7) significantly elevated (*p* < 0.01) compared to unaffected siblings (M = 41.9 ± 8.2). In contrast to the total data, individuals with PMD under 11 years old did not differ (M = 47.9 ± 9.6) from unaffected siblings. The individual sub-scales of bedtime resistance, daytime sleepiness and sleep duration were all significantly worse in participants with *SYNGAP1*-ID than unaffected siblings but did not significantly differ in PMD. Similar to the data from all ages, participants with either *SYNGAP-ID* or PMD who were less than 11 years old were significantly more likely to have parasomnias, night awakenings and sleep disordered breathing than unaffected siblings. Overall, these data demonstrate similar abnormalities for the *SYNGAP1*-ID participants under 11 years of age to the entire *SYNGAP1*-ID population of this study. In contrast, PMD participants under 11 years of age have fewer statistically significant differences in CSHQ sub-scales compared with unaffected siblings than the entire PMD study population did.

**Figure 3:**
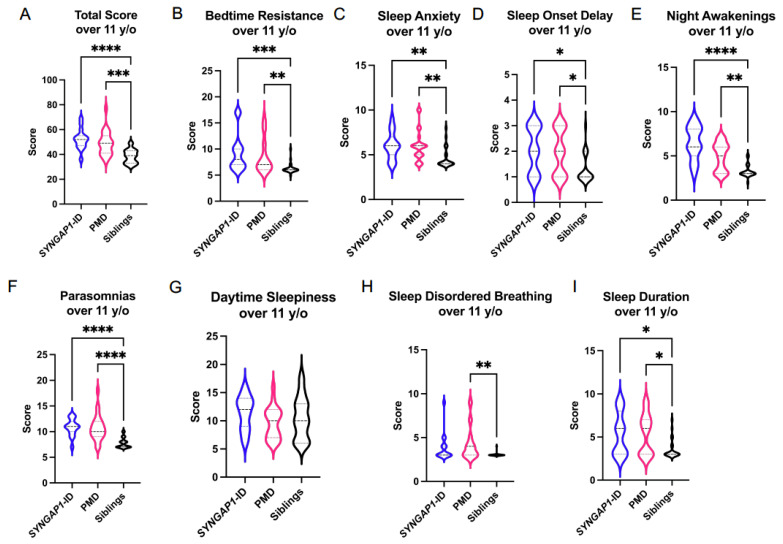
Total (**A**) and sub-scale scores for *SYNGAP1*-related intellectual disability (*SYNGAP1*-ID), Phelan–McDermid syndrome (PMD) and unaffected siblings (Siblings) 11 years of age and older. Sub-scales: (**B**) Bedtime Resistance, (**C**) Sleep Anxiety, (**D**) Sleep Onset Delay, (**E**) Night Awakenings, (**F**) Parasomnias, (**G**) Daytime Sleepiness, (**H**) Sleep Disordered Breathing, (**I**) Sleep Duration. Dunn’s multiple comparisons test * *p* < 0.05, ** *p* < 0.01, *** *p* < 0.001, **** *p* < 0.0001. Heavy dashed line is median, light dashed line is quartile.

We next evaluated the participants 11 years of age and over (Figure 3 and Table 4). The overall CSHQ score was significantly elevated in both the *SYNGAP1*-ID (M = 52.3 ± 8.2, *p* < 0.001) and PMD (M = 49.0 ± 7.4, *p* < 0.001) participants compared with unaffected siblings (M = 38.5 ± 5.5). Participants diagnosed with *SYNGAP1*-ID or PMD had significantly elevated scores in bedtime resistance, sleep anxiety, sleep-onset delay, night-time awakenings, parasomnias and sleep duration. PMD participants had an elevated sleep disordered breathing score not present in *SYNGAP1*-ID. Overall, these data demonstrate similar abnormalities for the *SYNGAP1*-ID participants under 11 years of age when compared to *SYNGAP1*-ID participants over 11 years old. In contrast, PMD participants under 11 years of age have fewer significant sleep phenotypes compared with PMD participants over 11 years old.

It is worthwhile highlighting the specific differences we identified between *SYNGAP1*-ID and PMD. The total score mean for *SYNGAP1*-ID was significantly higher than PMD for all ages and for subjects less than 11 years old (Figure 1A and Figure 2A). This increase in total score for *SYNGAP1*-ID was driven by significantly increased mean scores for bedtime resistance and daytime sleepiness. For the subjects less than 11 years of age, the elevated mean total score was driven by increased mean scores for sleep anxiety, daytime sleepiness and sleep duration.

We also asked parents of our participants if they were administering medications to improve sleep of their children. We found that 5 out of 61 (8%) of unaffected siblings took a sleep aid at least occasionally. Three unaffected siblings used melatonin and one used hydroxyzine. In contrast, 20 out of 64 participants (31%) with *SYNGAP1*-ID were given at least one sleep aid occasionally or nightly for their sleep disturbance revealing a significant difference from the unaffected siblings (X^2^ = 48.81, *p* < 0.0001). The sleep aids used by this population included melatonin (*n* = 13), guanfacine (*n* = 2), clonidine (*n* = 4), diphenhydramine (*n* = 1), trazodone (*n* = 3), aripiprazole (*n* = 1) and CBD oil (*n* = 3). For the PMD population, 15 out of 47 participants took at least one sleep aid (32%) revealing a similar increase in medication use compared with our unaffected siblings (X^2^ = 33.06, *p* < 0.0001). The medications used included melatonin (*n* = 9), clonidine (*n* = 4), clonazepam (*n* = 1), guanfacine (*n* = 1), lorazepam (*n* = 1), trazodone (*n* = 3) and zolpidem (*n* = 1).

## 4. Discussion

Sleep disturbances have previously been reported in clinical descriptions of synaptopathies including *SYNGAP1*-ID and PMD [10,11,12,13]. In this work, we evaluated sleep in these two populations using a standardized, well validated but simple to administer instrument, the Children’s Sleep Habits Questionnaire (CSHQ). We identified significant behavioral abnormalities for both populations including parasomnias, night-time awakenings and sleep onset delay. 

Overall, sleep scores were significantly worse in *SYNGAP1*-ID participants than PMD based upon higher total score and more sub-scales of the CSHQ with significant abnormalities in *SYNGAP1*-ID. The unique sleep abnormalities found only in *SYNGAP1*-ID included sleep anxiety and worse scores for sleep duration. 

Sleep issues are common in NDDs including autism and epilepsy [20]. All three disorders are thought to result in part from a failure of homeostatic synaptic plasticity [15], a form of synaptic plasticity that adjusts the strength of a neuron’s excitatory synapses up or down to stabilize firing, and is critical for controlling circuit hypo- or hyper-excitability. Sleep is proposed to promote scaling down of synaptic strengths thus priming synapses to respond to the awake state [16]. Impaired homeostatic scaling is seen in both SHANK3 and SynGAP, where knockdown of SHANK3 disrupts the ability of a neuron to adjust the strength of its excitatory synapses up while genetic knockdown of SynGAP leads to increase in the strength of excitatory synapses [15,17]. Hyper-excitability of neuronal circuits is potentially most relevant for *SYNGAP1* as >90% of individuals with pathogenic *SYNGAP1* mutations develop epilepsy and have abnormal interictal epileptiform discharges [10,12]. Moreover, epileptiform discharges have been found to be enhanced during sleep in both mice haploinsufficient for the murine orthologue of *SYNGAP1* and patients with pathologic mutations [21]. It has been hypothesized that abnormal epileptic discharges might disrupt normal sleep architecture leading to sleep disorders [22,23]. The enhanced night-time epileptiform discharges might explain the more severe sleep abnormalities observed in patients with *SYNGAP1* mutations and warrants further investigation.

We parsed out our data based upon age to determine if this is a critical variable to sleep abnormalities in synaptopathies. We chose to parse the data into two groups: those below 11 years of age and those 11 years of age and older to investigate changes in sleep abnormalities in pre-pubertal versus pubertal and post-pubertal individuals in these populations and because the CSHQ was originally validated in those less than 11 years of age [19]. We found that in general, total score and sub-scale scores were worse for individuals 11 years and over compared with those under 11 years of age. This was most striking for PMD where total CSHQ score was not significantly different in participants under 11 years of age but significantly elevated in those 11 years old and older compared to unaffected siblings.

No previous studies have systematically evaluated sleep in *SYNGAP1*-ID. In contrast, one previous study evaluated sleep abnormalities in PMD [18]. Similar to our study, Bro et al. determined that sleep abnormalities are common in individuals with PMD. However, the overall severity as measured by total CSHQ score (M = 51.7 ± 9.0) was greater than we observed. The reason for this discrepancy is unclear. The median age for participants with PMD in our study (9.0) was similar to this previous one (8.0). Bro et al. did not include unaffected siblings recruited contemporaneously with the PMD patients, instead relying on historical controls. As such, statistical analysis between PMD and unaffected siblings was not performed.

Both the *SYNGAP1*-ID and PMD populations were found to extensively use pharmacologic sleep aids, and more so than unaffected siblings. Some of the sleep aids used by these populations are also used for other indications in children with neurodevelopmental disabilities, such as the alpha-2 agonists clonidine and guanfacine which are sometimes used for hyperactivity and aggressive behaviors. Thus, some of these medications might have been given for duel purposes.

This study has several limitations. First, we opted to recruit all individuals with *SYNGAP1*-ID and PMD we could, regardless of age, due to the rarity of these disorders. Thus, some of the participants were outside of the validated age range for the CSHQ. Second, the majority of this data was collected during the COVID-19 pandemic which disrupted schedules worldwide and likely influenced some of the results. Finally, there was a significant difference in the average ages of individuals with PMD and unaffected siblings.

Development of targeted treatments for NDDs caused by genetic abnormalities has been slow to be realized. The reason for the difficulty in their development is likely multi-factorial with one cause being lack of quantitative clinical endpoints that are responsive to intervention in the timeline of typical clinical trials. Improvement in sleep represents one potential clinical endpoint for synaptopathies. Studies such as this one can provide the baseline natural history data needed for future clinical trials for these disorders.

## 5. Conclusions

Utilizing a structured sleep assessment tool, the Childhood Sleep Habits Questionnaire (CSHQ), we discovered that children with two neurodevelopmental disorders due to mutations in synaptic protein encoding genes, Phelan-McDermid syndrome (PMD) and *SYNGAP1*-related Intellectual Disability (*SYNGAP1*-ID) have significant sleep disturbances. For both disorders, night awakenings and parasomnias were significantly worse in the total populations as well as in both age ranges (less than 11 years and 11 years old and greater) in which the data was analyzed. Participants with *SYNGAP*-ID had significantly worse scores in bedtime resistance and daytime sleepiness than PMD participants. Due to their sleep disturbances, pharmacologic sleep aids were commonly prescribed for both neurodevelopmental disorders. We propose that sleep disturbances are valid clinical endpoints for clinical trials of targeted therapies for these neurodevelopmental disorders.

## Figures and Tables

**Table 1:** Demographics, mean (standard deviation).

	*SYNGAP1*-ID (*n* = 64)	PMD (*n* = 47)	Siblings (*n* = 61)	Kruskal-Wallis Statistic	Kruskal-Wallis Test (*p*-Value)
**Age**	8.4 (8.2)	12.7 (9.2)	9.2 (4.5)	10.12	0.0063
**Age (under 11)**	5.6 (2.4)	5.6 (2.5)	6.1 (2.5)	0.88	0.6453
**Age (11 and over)**	17.4 (13.1)	20.2 (7.5)	13.9 (2.0)	17.38	0.0002
**Males**	48%	49%	41%		0.63 *
**Females**	52%	51%	59%		0.63 *

* Chi-square = 0.93; df = 2

**Table 2:** Combined data from CSHQ, mean (standard deviation).

	*SYNGAP1*-ID (*n* = 64)	PMD (*n* = 47)	Siblings (*n* = 61)	Kruskal-Wallis Statistic	Kruskal-Wallis Test (*p*-Value)
**Total Score**	52.2 (7.7)	47.6 (9.2)	40.5 (7.4)	51.65	<0.0001
**Bedtime Resistance**	10.5 (3.1)	8.7 (3.2)	7.3 (2.1)	35.74	<0.0001
**Sleep Anxiety**	6.7 (1.7)	5.9 (1.9)	5.2 (1.6)	22.82	<0.0001
**Sleep Onset Delay**	1.8 (0.8)	1.8 (0.8)	1.4 (0.6)	12.99	0.0015
**Night Awakenings**	6.0 (1.8)	5.1 (1.7)	3.7 (1.3)	46.48	<0.0001
**Parasomnias**	10.6 (1.9)	10.9 (2.1)	8.3 (1.6)	53.57	<0.0001
**Daytime Sleepiness**	11.2 (2.7)	9.0 (2.8)	10.3 (3.5)	17.14	0.0002
**Sleep Disordered Breathing**	3.6 (1.0)	4.1 (1.7)	3.2 (0.7)	22.72	<0.0001
**Sleep Duration**	5.4 (2.0)	4.8 (2.0)	3.6 (1.1)	31.00	<0.0001

**Table 3:** CSHQ data under 11 years old, mean (standard deviation).

	*SYNGAP1*-ID (*n* = 49)	PMD (*n* = 24)	Siblings (*n* = 37)	Kruskal-Wallis Statistic	Kruskal-Wallis Test (*p*-Value)
**Total Score**	52.2 (7.7)	46.2 (8.9)	41.9 (8.2)	27.55	<0.0001
**Bedtime Resistance**	10.9 (2.9)	9.6 (3.1)	8.3 (2.4)	20.33	<0.0001
**Sleep Anxiety**	6.9 (1.8)	5.8 (2.0)	5.6 (1.8)	12.09	0.0024
**Sleep Onset Delay**	1.8 (0.8)	1.6 (0.7)	1.4 (0.7)	4.99	0.0825
**Night Awakenings**	5.8 (1.8)	5.3 (1.9)	4.1 (1.5)	19.76	<0.0001
**Parasomnias**	10.5 (2.0)	11 (1.7)	8.7 (1.9)	25.79	<0.0001
**Daytime Sleepiness**	11.1 (2.6)	8.5 (2.7)	9.9 (3.4)	17.37	0.0002
**Sleep Disordered Breathing**	3.6 (0.9)	3.9 (1.5)	3.3 (0.9)	9.58	0.0083
**Sleep Duration**	5.4 (1.9)	4.3 (1.7)	3.6 (1.2)	23.96	<0.0001

**Table 4:** CSHQ data 11 years and older, mean (standard deviation).

	*SYNGAP1*-ID (*n* = 15)	PMD (*n* = 23)	Siblings (*n* = 24)	Kruskal-Wallis Statistic	Kruskal-Wallis Test (*p*-Value)
**Total Score**	52.3 (8.2)	49.0 (9.6)	38.5 (5.5)	25.81	<0.0001
**Bedtime Resistance**	9.2 (3.6)	8.6 (3.2)	6.3 (1.0)	18.14	0.0001
**Sleep Anxiety**	6.0 (1.5)	6.0 (1.7)	4.6 (1.1)	14.07	0.0009
**Sleep Onset Delay**	2.1 (0.9)	2.0 (0.9)	1.3 (0.6)	9.88	0.0072
**Night Awakenings**	6.5 (1.9)	5.0 (1.6)	3.3 (0.7)	27.54	<0.0001
**Parasomnias**	10.8 (1.7)	10.8 (2.5)	7.8 (1.0)	28.14	<0.0001
**Daytime Sleepiness**	11.4 (3.0)	9.6 (2.8)	10.5 (3.7)	3.234	0.1984
**Sleep Disordered Breathing**	3.9 (1.6)	4.3 (1.9)	3.0 (0.2)	12.94	0.0015
**Sleep Duration**	5.4 (2.4)	5.4 (2.3)	3.6 (1.1)	9.109	0.0105

## Data Availability

Data is available from the authors upon reasonable request.

## Supplementary Materials

The following are available online at https://www.mdpi.com/article/10.3390/brainsci11091229/s1, Table S1: Scores of sex impacted on sleep disorders.
