# Supplementary material for: Sleep Abnormalities in the Synaptopathies—SYNGAP1-Related Intellectual Disability and Phelan–McDermid Syndrome
Source: Brain Sci. 2021 Sep 17;11(9):1229. doi: 10.3390/brainsci11091229 (PMC8472329; doi:10.3390/brainsci11091229)
Supplement: Supplementary file 1 [file brainsci-11-01229-s001.zip › brainsci-1356955-supplementary.pdf]

Supplemental Table S1. Scores of sex impacted on sleep disorders.

	Mean	S.D.	Mann-Whitney U	*p* Value
***SYNGAP1*-ID male**	50.4	8.5	376	0.07
***SYNGAP1*-ID female**	53.8	6.6		
**PMD male**	48.3	10.5	264.5	0.81
**PMD female**	46.8	8.1		
**Siblings male**	38.6	6.1	339.5	0.11
**Siblings female**	41.9	8.0		
